# Isolation, phenotypic characterization and genome wide analysis of a *Chlamydomonas reinhardtii* strain naturally modified under laboratory conditions: towards enhanced microalgal biomass and lipid production for biofuels

**DOI:** 10.1186/s13068-017-1000-0

**Published:** 2017-12-22

**Authors:** Sung-Eun Shin, Hyun Gi Koh, Nam Kyu Kang, William I. Suh, Byeong-ryool Jeong, Bongsoo Lee, Yong Keun Chang

**Affiliations:** 1 Department of Chemical and Biomolecular Engineering, KAIST, 291 Daehak-ro, Yuseong-gu, Daejeon, 34141 Republic of Korea; 2 Advanced Biomass R&D Center, 291 Daehak-ro, Yuseong-gu, Daejeon, 34141 Republic of Korea; 3 Present Address: LG Chem, 188 Munji-ro, Yuseong-gu, Daejeon, 34122 Republic of Korea

**Keywords:** Microalgae, *Chlamydomonas reinhardtii*, Adaptive evolution, Fatty acid methyl ester, Biodiesel, Nitrogen starvation

## Abstract

**Background:**

Microalgal strain development through genetic engineering has received much attention as a way to improve the traits of microalgae suitable for biofuel production. However, there are still some limitations in application of genetically modified organisms. In this regard, there has been recent interest in the isolation and characterization of superior strains naturally modified and/or adapted under a certain condition and on the interpretation of phenotypic changes through the whole genome sequencing.

**Results:**

In this study, we isolated and characterized a novel derivative of *C. reinhardtii*, whose phenotypic traits diverged significantly from its ancestral strain, *C. reinhardtii* CC-124. This strain, designated as CC-124H, displayed cell population containing increased numbers of larger cells, which resulted in an increased biomass productivity compared to its ancestor CC-124. CC-124H was further compared with the CC-124 wild-type strain which underwent long-term storage under low light condition, designated as CC-124L. In an effort to evaluate the potential of CC-124H for biofuel production, we also found that CC-124H accumulated 116 and 66% greater lipids than that of the CC-124L, after 4 days under nitrogen and sulfur depleted conditions, respectively. Taken together, our results revealed that CC-124H had significantly increased fatty acid methyl ester (FAME) yields that were 2.66 and 1.98 times higher than that of the CC-124L at 4 days after the onset of cultivation under N and S depleted conditions, respectively, and these higher FAME yields were still maintained by day 8. We next analyzed single nucleotide polymorphisms (SNPs) and insertion/deletions (indels) based on the whole genome sequencing. The result revealed that of the 44 CDS region alterations, 34 resulted in non-synonymous substitutions within 33 genes which may mostly be involved in cell cycle, division or proliferation.

**Conclusion:**

Our phenotypic analysis, which emphasized lipid productivity, clearly revealed that CC-124H had a dramatically enhanced biomass and lipid content compared to the CC-124L. Moreover, SNPs and indels analysis enabled us to identify 34 of non-synonymous substitutions which may result in phenotypic changes of CC-124H. All of these results suggest that the concept of adaptive evolution combined with genome wide analysis can be applied to microalgal strain development for biofuel production.

**Electronic supplementary material:**

The online version of this article (10.1186/s13068-017-1000-0) contains supplementary material, which is available to authorized users.

## Background

Petroleum has been considered to be an irreplaceable source for the production of fuels and chemical materials since the industrial revolution. However, continuous use of fossil fuels has caused many environmental problems and the exhaustion of oil deposits. These problems have raised the necessity to develop new renewable energy sources [1]. In this respect, microalgae have been in the spotlight as a promising feedstock for biofuels and value-added products due to their high lipid contents and rapid growth through photosynthesis consuming CO_2_ more efficiently compared to other feedstocks, such as crops and lignocellulosic biomass [1–3].

Among the various microalgae species, green unicellular microalgae, *Chlamydomonas reinhardtii*, has been intensively studied for over 30 years as a model for basic physiology and applied biotechnology research [4]. Studies in this organism have provided tremendous knowledge for understanding the mechanism behind algal metabolism and for developing molecular tools and techniques for genetic engineering. These information and tools from *C. reinhardtii* are now being applied to other microalgae as well to maximize biomass and lipid productivity [4–7]. In particular, current efforts have emphasized the production of triacylglycerols (TAGs) that can be used as a substrate for biodiesel production [8]. Since the identification and characterization of starchless mutants in *C. reinhardtii*, several studies have shown that wild-type (WT) and mutant strains accumulate a significant amount of neutral lipids (TAGs) in response to nitrogen, sulfur or salt stress [9–14]. Meanwhile, there have been other efforts to increase biomass through the genetic engineering of genes which are involved in the photosynthesis of *C. reinhardtii*. The truncated light harvesting antenna complex (*tla*) mutants have shown an increased biomass compared to their WT strain [15–19]. These previous studies suggest that various traits of microalgae including growth and lipid synthesis can be improved through genetic engineering.

However, despite these potentials, there are still some limitations in the use of GMOs and the development of microalgal strains that meet academic and industrial standards for successful biofuel production. These limitations sometimes include genetic redundancy, controversial phenotypes and unstable expression of introduced genes in the genetically engineered strains. Thus far, the most common strategy for enhancing lipid production is likely to express one of the genes involved in TAG synthesis. Unfortunately, however, much greater accumulation of TAG has been rarely guaranteed with this strategy. For instance, targeted overexpression of type 2 diacylglycerol acyltransferases (DGATs) DGTT1, DGTT2 and DGTT3 designated as DGAT2-a, DGAT2-c and DGAT2-b, respectively, did not affect TAG accumulation in *C. reinhardtii* [20], while overexpression of DGTT1 and DGTT3 designated as DGAT2-5 and DGAT2-1 led to increased TAG accumulation [21]. This indicates that not only it is difficult to expect increased TAG accumulation even with overexpression of one gene which plays an important role in TAG biosynthesis, but also different phenotypes can be exhibited even in strains modified with the same target genes. In addition to these problems, unstable expression in transgenic strains has long been reported in higher plants and *C. reinhardtii* [22–25]. This may ultimately result in the loss of phenotypes making it difficult for researchers to use genetically engineered strains for biofuel production.

Microalgae are ubiquitous microorganisms present in almost all the ecosystems implying that microalgae may have naturally developed their favorable traits through environmental adaptations [7]. In this context, strain development of *C. reinhardtii* through experimental adaptive evolution has succeeded in its efforts to improve their growth, lipid content, photosynthetic efficiency, CO_2_ utilization efficiency and heterotrophy, respectively [26–29]. Moreover, single nucleotide polymorphisms (SNPs) or insertions/deletions (indels) analysis using progressive and large-scale sequencing called next-generation sequencing (NGS) technologies have provided new insights for the identification of sequence variations within the individual genomes and for the interpretation of mutant phenotypes [26, 30–34]. These suggest that an adaptive evolution strategy combined with NGS technology could be an alternative method to circumvent the regulations on the use of GMOs and the current limitations of genetic engineering for microalgal strain development.

One major difference between the natural adaptive evolution and intentional adaptive evolution is the presence and absence of selection pressure. While the presence of selection pressure that comes with intentional adaptive evolution may increase the likelihood of obtaining a strain with desired characteristics, there is also a greater probability for the culture to lose that desired trait if that selection pressure is removed, as the wild-type strain may regain fitness advantage against the mutant. In this study, we isolated naturally, but not intentionally adapted *C. reinhardtii* CC-124 during cell maintenance under laboratory conditions. The phenotypic traits of this strain designated as CC-124H were visibly distinct from its ancestral WT strain. To characterize the phenotypic changes in CC-124H and to further assess the potential application of CC-124H for biofuel production, we analyzed the biomass, FAME contents and biochemical components of the WT and CC-124H strains under normal and nutrient stress conditions such as N and S deprivation. In addition, we performed whole genome sequencing and SNPs/indels analysis to identify sequence variations and to understand phenotypic changes in the CC-124H strain.

## Results

### Isolation of the CC-124H strain

**Fig. 1:**
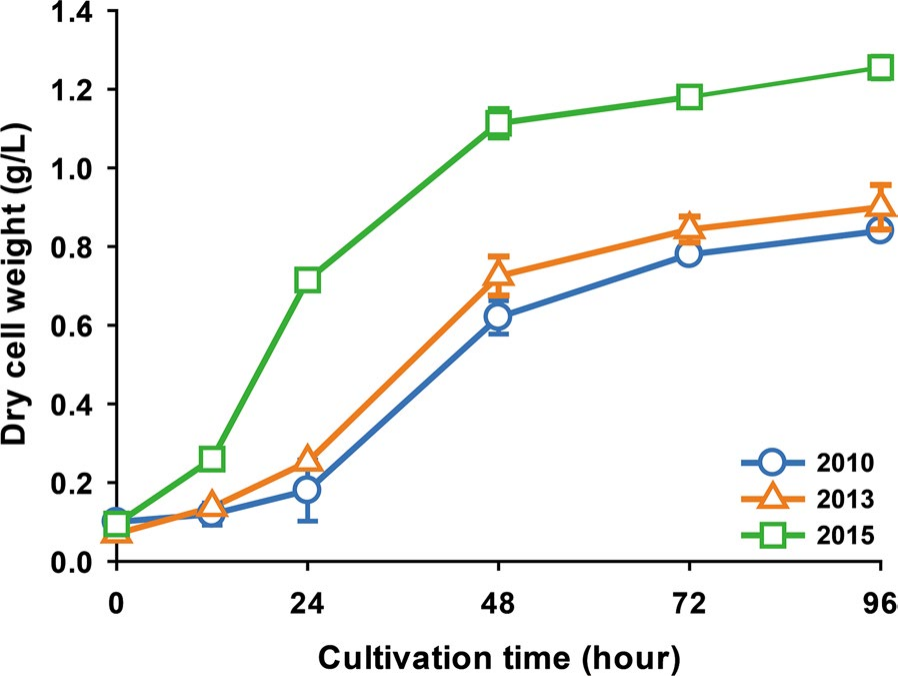
Growth comparison of *C. reinhardtii* in year 2010, 2013, and 2015. Cells were cultivated in liquid TAP medium. Data are expressed as ± SD (*n* = 3)

During the process of maintaining the *C. reinhardtii* CC-124 strain, in which colonies were regularly transferred onto fresh TAP agar plates once every month for approximately 4 years and incubated at 25 °C under a continuous fluorescent light condition (120 µmol photons/m^2^/s), we found that the growth rate of the *C. reinhardtii* CC-124 strain became faster compared to the last 4 years. When the initial cell concentration of the inoculum was adjusted to 0.1 OD_750_, the dry cell weight (DCW) of the *C. reinhardtii* CC-124 strain measured in 2015 reached 0.72 g/L after 24 h of growth, which was about three and four times greater than those measured in 2013 and 2010, respectively (Fig. 1). Moreover, the specific growth rate during the exponential phase but not the stationary phase was more rapid (Additional file 1: Table S1), indicating that the physiological phenotype of the *C. reinhardtii* CC-124 strain, especially for growth related to biomass production, may have been naturally altered under laboratory conditions over the 4 years. The mutant had also exhibited loss of mobility, no flagella, and lacks the negative phototaxis that is normally found in the wild-type cells, as well as substantial increase of the proportion of cells in the palmelloid state. To further understand the physiological change and evaluate the potential as a source for biofuel production, a *C. reinhardtii* CC-124 strain with a better trait for biomass production was isolated and designated as CC-124H. For the subsequent experiment, CC-124H was compared with the wild-type strain that was kept for long-term storage in slant culture tubes under low light condition, designated CC-124L.

### Growth of CC-124H under normal conditions

**Fig. 2:**
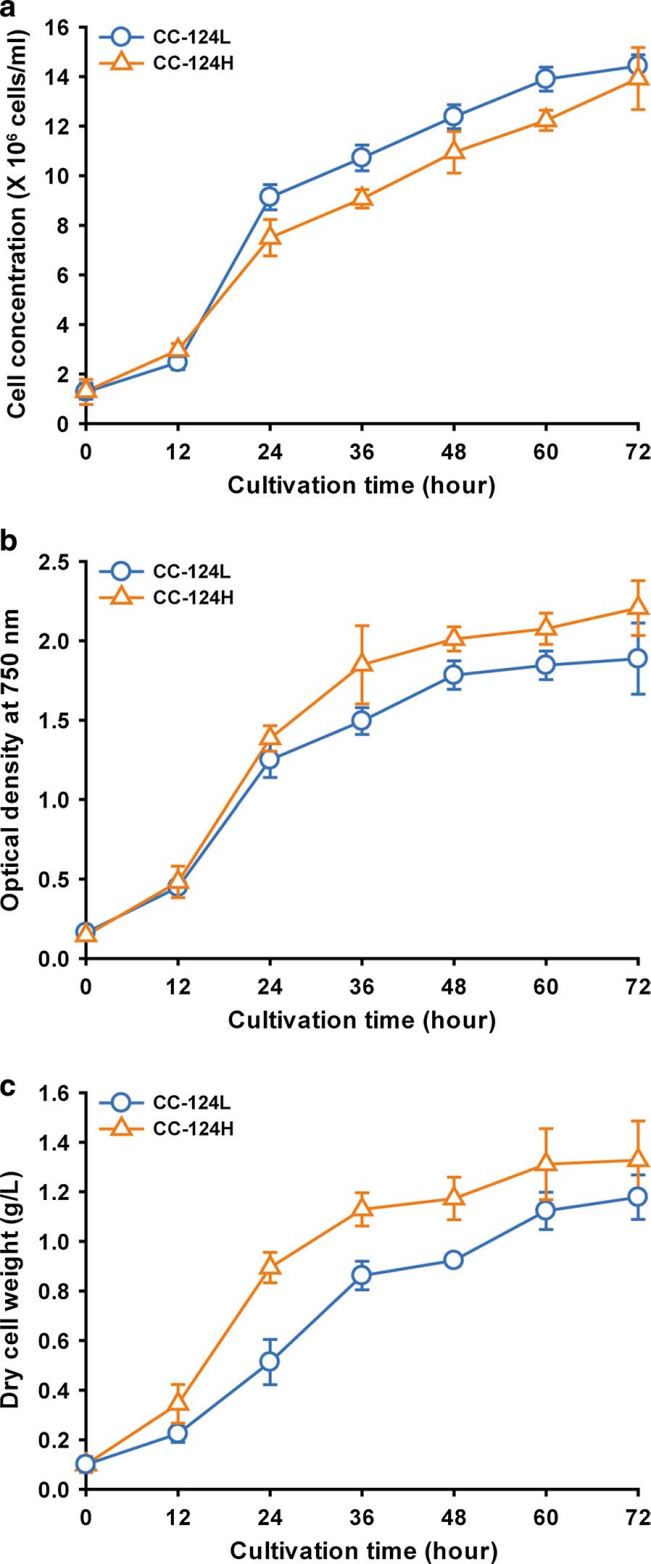
Growth analyses of CC-124H in liquid TAP medium. Growth curves based on cell density (**a**), optical density at 750 nm (**b**), and dry cell weight (**c**) were obtained during 72 h. Data are expressed as ± SD (*n* = 4)

We first analyzed the growth traits of CC-124L and CC-124H, including cell number, OD_750_ and DCW under normal conditions. Our results revealed that the growth rate of CC-124H based on the OD_750_ and DCW was continuously higher than that of the CC-124L over the whole cultivation, while the growth rate based on cell number was lower (Fig. 2a–c). In particular, the DCW of CC-124H at 24 and 72 h was approximately 74 and 13% greater than that of the CC-124L, respectively (Fig. 2c). Moreover, the specific growth rate of CC-124H based on the DCW at the early period (from 0 to 24 h) was 1.34 times higher compared with the CC-124L (Table 1). To investigate the reason behind the increased biomass accumulation in CC-124H, we next measured the cell size at 24 h representing the exponential phase and at 72 h representing the stationary phase (Table 1 and Additional file 2: Figure S1). The result showed that CC-124H displays a population with average particle size measurements that were 30 and 26% bigger than that of CC-124L on average at 24 and 72 h, respectively. We also analyzed the nutrient consumption rate in the CC-124L and CC-124H to understand how nutrient uptake is associated with their proliferation and biomass production (Table 1). It should be noted that even though the cell number of CC-124H was constantly lower than that of the CC-124L during the cultivation, the growth rate based on cell counting remained the same as the CC-124L except between 12 and 24 h (Fig. 2a). Our nutrient analysis revealed that the average consumption rate of nutrients including acetate, NH_4_^+^ and PO_4_^2−^ in CC-124H was at the same level as the CC-124L. Interestingly, however, CC-124H utilized much more acetate as a carbon source and NH_4_^+^ as a nitrogen source by 24 h compared with the CC-124L (Table 1). This may be interpreted as CC-124H used much more carbon and nitrogen source for efficient proliferation and maintenance of the larger cells especially at the exponential phase based on the experimental evidence that the population of CC-124H at the exponential phase contains much bigger cells compared with that at the stationary phase (Additional file 2: Figure S1). Taken together, these results suggest that the newly isolated CC-124H has significantly distinct phenotypes apart from the CC-124L.

**Table 1:** Specific growth rate, cell size, and nutrient consumption rate of CC-124L and CC-124H under normal condition

Strain	Specific growth rate (mg/mL/h)^a^		Cell size (μm)^b^		Average rate of nutrient consumption (ppm/h)^c^					
					0–24 h			0–72 h		
	0–24 h	24–72 h	24 h	72 h	*C*	*N*	*P*	*C*	*N*	*P*
CC-124L	0.070 ± 0.015	0.018 ± 0.003	7.16 ± 0.01	7.30 ± 0.01	22.90	2.47	1.39	13.90	1.34	1.20
CC-124H	0.094 ± 0.014*	0.008 ± 0.001**	9.36 ± 0.11	9.24 ± 0.02	33.10	3.20	1.14	13.90	1.32	0.77

Data are expressed as ± SD (*n* = 4). Significant differences, as determined by Student’s *t* test, are indicated by asterisk (* *P* < 0.05, ** *P* < 0.01, *** *P* < 0.001)

^a^Specific growth rate based on dry cell weight was calculated as following equations: ${\text{Specific growth rate }}\left( {\upmu/{\text{day}}} \right) = { \ln }\left( {X_{ 2} /X_{ 1} } \right)/\left( {T_{ 2} - T_{ 1} } \right),$ where *X*_1_ and *X*_2_ are the initial and final dry cell weight, and *T*_1_ and *T*_2_ are the initial and final times

^b^Cell size measured among the 50% in the middle value

^c^Nutrient consumption was based on acetate for *C*, ammonium for *N*, and phosphate for *P* sources

### Biomass and FAME analyses of CC-124H under nitrogen and sulfur starvation

**Fig. 3:**
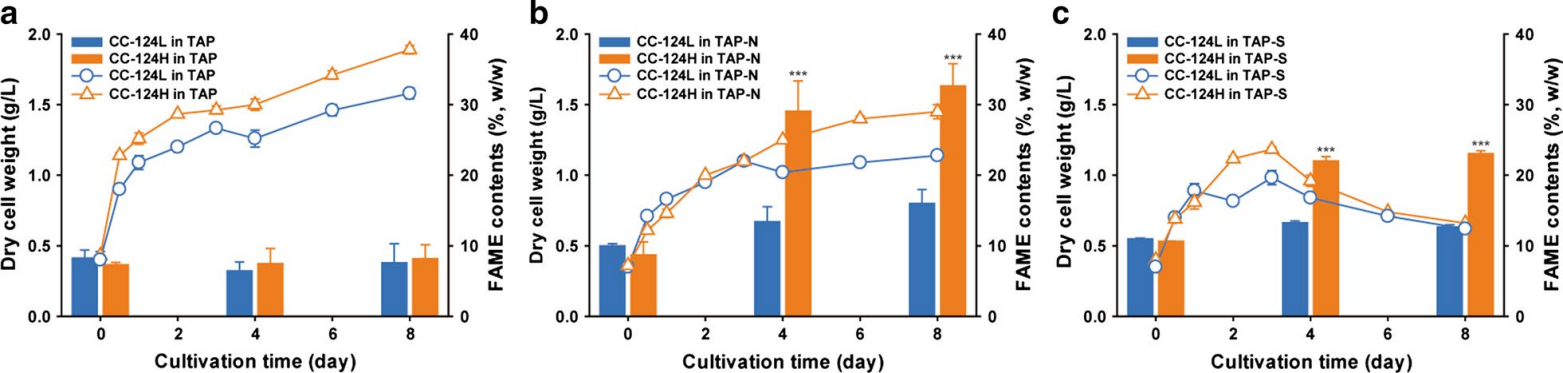
Biomass and lipid production of CC-124H under different nutrient starved conditions. Cells were cultivated under normal conditions (**a**), N starvation (**b**), and S starvation (**c**). Lines and bars each indicate the DCW and the FAME contents at different cultivation conditions. Data are expressed as ± SD (*n* = 4). Significant differences, as determined by Student’s *t* test, are indicated by asterisk (*P < 0.05, **P < 0.01, ***P < 0.001)

**Fig. 4:**
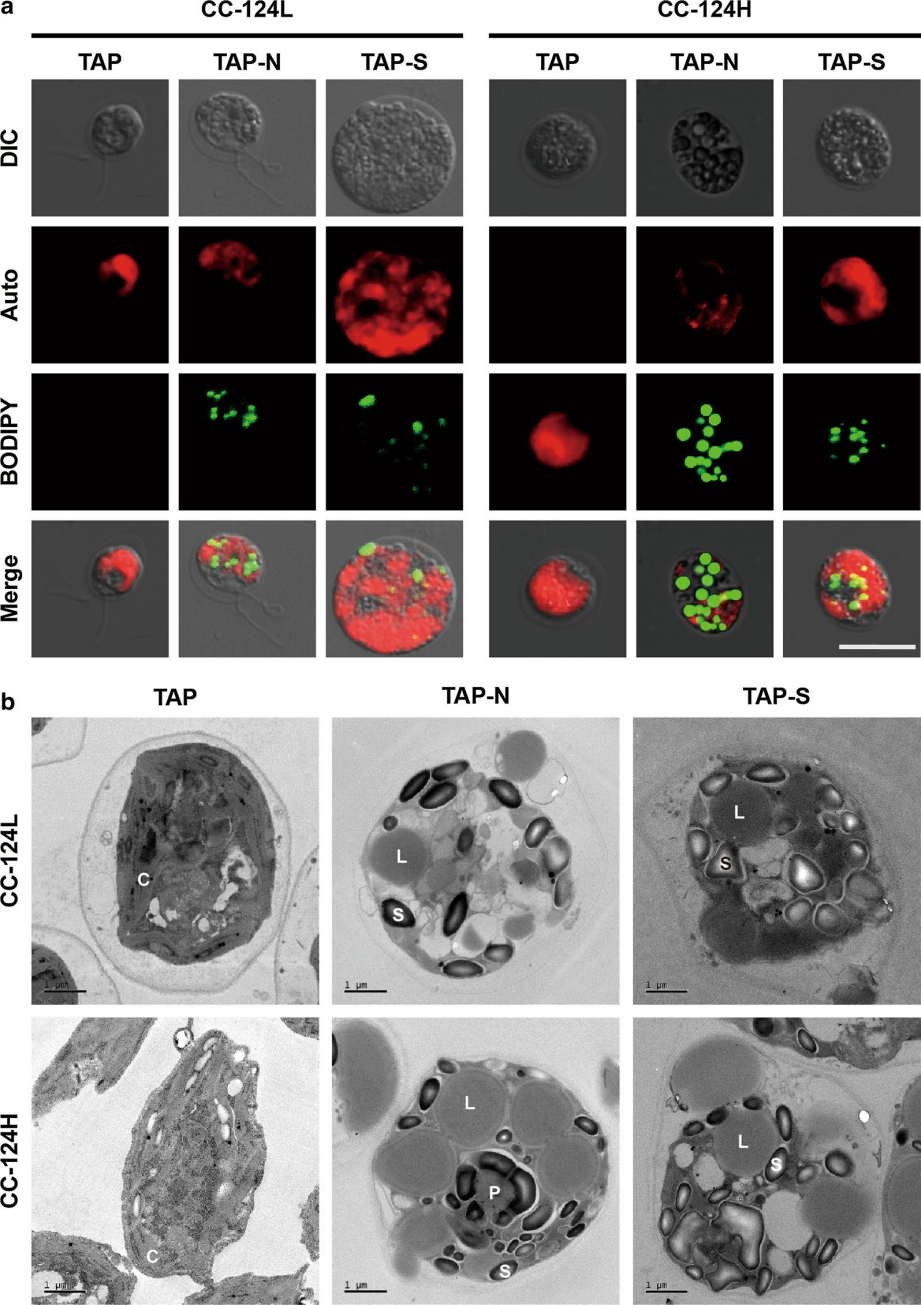
Accumulated lipid droplets of CC-124H after 4 days of nutrient starved conditions. **a** Confocal microscopy images stained with BODIPY 505/515. 1st row: differential interference contrast (DIC); 2nd row: chlorophyll autofluorescence, 3rd row: BODIPY fluorescence, and 4th row: all three images merged are shown. The scale bar indicates 10 μm. **b** Transmission electron microscopy images. *C* chloroplast, *L* lipid droplet, *S* starch granule, *P* pyrenoid. The scale bars indicate 1 μm

To assess if CC-124H can be used as a biomass source for biofuel production, we analyzed the biomass and FAME of the CC-124L and CC-124H under nitrogen (N) or sulfur (S) starvation conditions (Fig. 3 and Table 2), because previous studies have shown that *C. reinhardtii* can accumulate a significant amount of lipids in response to N or S deprivation [12–14]. Cultivation of the CC-124L and CC-124H under N or S starvation conditions was performed by following modified protocols from previous studies [13, 35]. Briefly, cells grown to exponential phase were harvested and washed twice with the appropriate TAP medium depending on the culture conditions (TAP, TAP without N and TAP without S). Then, the cells whose OD_750_ was approximately 0.7 were inoculated into the TAP medium without N and S, respectively. In agreement with the previous studies, lipids in both the CC-124L and CC-124H were induced in response to the N and S deprivation (Fig. 3). Interestingly, however, CC-124H had a significant increase in FAME contents that was approximately twofold higher than that of CC-124L at both 4 and 8 days after the onset of cultivation under N and S deprivation conditions, respectively (Fig. 3b, c), while additive FAME production was not induced under normal conditions over the cultivation (Fig. 3a). This implies that the lipid accumulation mechanism of CC-124H in response to N or S depletion was likely altered from CC-124L. We also analyzed the biomass production of CC-124L and CC-124H (Fig. 3). It should be noted that nutrient stress conditions including N and P sources result in a low level of mass production, and biomass production is inversely proportional with the amount of lipid contents, because cells use a lot of energy for growth events such as cell division rather than lipid accumulation [36]. However, interestingly, our biomass analysis result revealed that the DCW of CC-124H at 8 days was 30% greater than that of CC-124L even under N starvation (Fig. 3b), whereas there was no significant difference in biomass production under S starvation condition after 4 days of cultivation (Fig. 3c). Eventually, the higher biomass production combined with the increased FAME contents of CC-124H in response to N deprivation resulted in a much higher FAME yield that was 2.66 and 2.65 times greater than that of the CC-124L after 4 and 8 days under N depleted conditions, respectively (Table 2). To elucidate the highly induced lipid accumulation of CC-124H in vivo, we next performed microscopic analysis which is able to visualize lipid bodies accumulated in response to N or S starvation (Fig. 4). Consistent with the FAME result, our analysis revealed that the CC-124H cells under N and S depleted conditions, especially grown in TAP medium without N source, clearly had much bigger and/or numerous lipid bodies compared to the CC-124L cells grown in normal conditions, when observing both under a confocal microscope and transmission electron microscope (TEM) (Fig. 4a, b). To sum up, the results from our biomass and FAME analyses clearly revealed that CC-124H can produce a large amount of lipids as well as biomass compared with the CC-124L and further suggest that more studies on CC-124H may provide us important insights to elucidate the mechanisms behind the relationship between lipid accumulation and growth under N depleted conditions as well as normal conditions.

**Table 2:** Biomass and FAME of CC-124H under nutrient starvation condition

Time (day)	Culture	CC-124L			CC-124H		
		Biomass (g/L)	FAME contents (%)	FAME yield (mg/L)	Biomass (g/L)	FAME contents (%)	FAME yield (mg/L)
0	TAP	0.38 ± 0.03	8.26 ± 1.13	32.29 ± 5.15	0.41 ± 0.04	7.30 ± 1.74	29.98 ± 9.15
	TAP-N	0.38 ± 0.03	10.05 ± 0.31	36.25 ± 3.48	0.39 ± 0.03	9.10 ± 1.76	34.95 ± 9.04
	TAP-S	0.37 ± 0.02	10.97 ± 0.13	40.59 ± 2.71	0.39 ± 0.02	10.63 ± 0.03	41.97 ± 2.25
4	TAP	1.19 ± 0.09	6.44 ± 1.26	80.13 ± 18.12	1.42 ± 0.09**	7.46 ± 0.92	108.88 ± 16.39*
	TAP-N	0.97 ± 0.05	14.15 ± 2.24	141.04 ± 20.16	1.22 ± 0.04***	30.50 ± 4.42***	374.54 ± 51.72**
	TAP-S	0.82 ± 0.02	13.24 ± 0.29	108.63 ± 4.79	0.98 ± 0.03***	22.01 ± 0.60***	214.79 ± 12.27***
8	TAP	1.46 ± 0.12	7.58 ± 2.73	115.38 ± 38.74	1.74 ± 0.15*	8.18 ± 1.79	147.58 ± 29.41
	TAP-N	1.11 ± 0.04	16.14 ± 1.68	180.35 ± 15.69	1.44 ± 0.04***	33.49 ± 3.16***	477.89 ± 36.43***
	TAP-S	0.57 ± 0.05	12.66 ± 0.32	72.74 ± 5.92	0.64 ± 0.03	23.07 ± 0.41***	150.15 ± 7.07***

Data are expressed as ± SD (*n* = 4). Significant differences, as determined by Student’s *t* test, are indicated by asterisk (* *P* < 0.05, ** *P* < 0.01, *** *P* < 0.001)

### Carbohydrate, protein and chlorophyll quantification

**Fig. 5:**
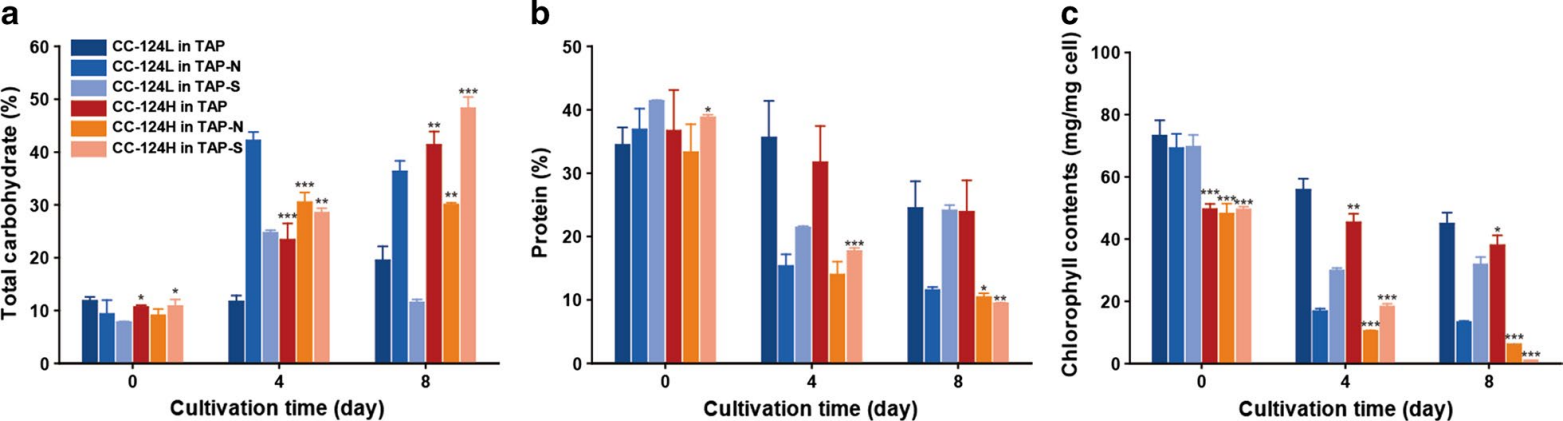
Biochemical composition and chlorophyll content changes of CC-124H under N and S starvation. Total carbohydrate (**a**), protein (**b**) and chlorophyll (**c**) contents were measured at 0, 4, and 8 days of induction. Data are expressed as ± SD (*n* = 4). Significant differences, as determined by Student’s *t* test, are indicated by asterisk (*P < 0.05, **P < 0.01, ***P < 0.001)

It has long been proposed that photosynthetic microalgae fix carbon, and these photosynthetically fixed carbons are transformed into major reserve macromolecules such as lipids, carbohydrates and proteins through a carbon assimilation and partitioning mechanism [37]. To further understand the mechanism underlying the significantly increased lipids in CC-124H and the corresponding changes of the biochemical compositions, we also analyzed the carbohydrate, protein and chlorophyll levels of the CC-124L and CC-124H grown under N and S depleted conditions, respectively (Fig. 5). Our results showed that CC-124H accumulated greater quantities of carbohydrates more rapidly compared to that of CC-124L during cultivation under both normal and S depleted conditions. Interestingly, however, CC-124H accumulated carbohydrates to a much lesser extent under N depleted conditions compared to CC-124L, which still contained a significant amount of carbohydrates under the same conditions. Finally, both the protein and chlorophyll contents were found to be decreased when the cultures were subjected to N and S depleted conditions as expected. However, CC-124H showed far lower levels of protein and chlorophyll contents compared to that of the CC-124L in the N and S depleted media. In particular, the extent of reduction in chlorophyll contents was particularly striking in the case of CC-124H under the S depleted conditions, because the culture was nearly devoid of chlorophyll and white in appearance (Fig. 5).

### Whole genome sequencing and SNPs/indels analysis

**Fig. 6:**
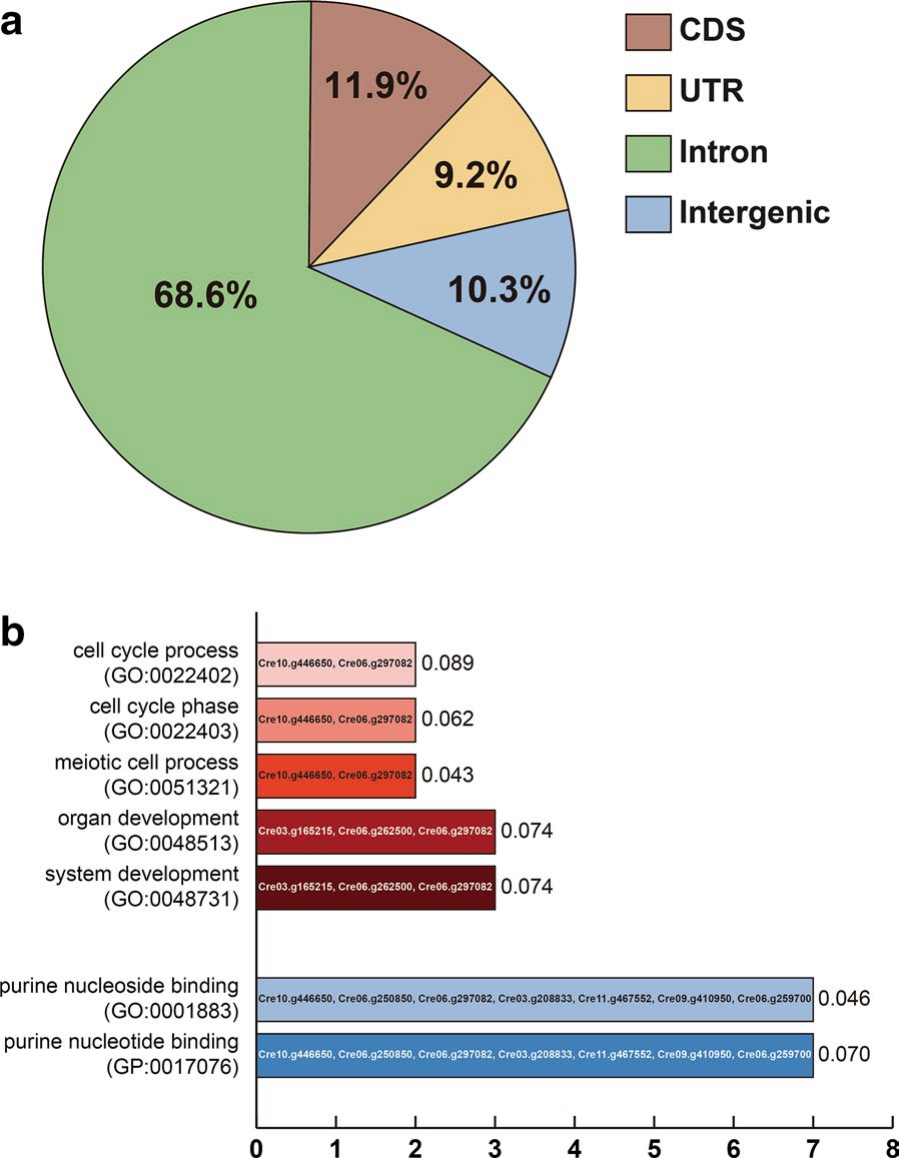
SNP/indels and GSEA of CC-124H **a** the locational pattern of single polymorphisms (SNPs) and insertion/deletion (indels) between CC-124H and CC-124L. **b** GSEA (GSEA) analysis by gene ontology (GO) depth 3. 19 non-synonymous genes in CDS regions were annotated by TAIR10 database, and the sensitivity test was conducted by gene set enrichment (GSEA) methods. Nine annotated genes remained, and the *P* value for each gene was calculated by Fisher’s exact test. The *P* value, GO term and phytozome locus name of *C. reinhardtii* were indicated in each column. The top five columns belong to the biological process domain, which are displayed in red colors. The below two columns in blue colors are molecular function domain

To investigate the variation of CC-124H in a genome level and understand the significant phenotypic changes of CC-124H in biomass and FAME production, we performed whole genome sequencing and SNPs/indels analysis of the CC-124L and CC-124H. A schematic workflow of the genome sequencing and SNPs/indels analysis is summarized in the Additional file 3: Figure S2. After filtering was done to remove low-quality sequence, the average coverage of the genome was 25.95× and 31.03× in the CC-124L and CC-124H, respectively. We obtained approximately 95% of the mapping region in both CC-124L and CC-124H sequencing data after alignment to the *C. reinhardtii* reference genome v5.5 (Phytozome ver 10.1). Our analysis revealed that there were 29,222 and 28,599 SNPs/indels in the CC-124L and CC-124H, respectively, when compared to the reference genome. In an effort to identify polymorphisms and indels specific to CC-124H, we found a total of 369 SNPs/indels comprising of 256 SNPs and 113 indels against the CC-124L. For the 369 SNPs/indels, 90% of them (331 SNPs/indels) occurred in genic regions which can possibly affect the function of proteins as follows: 253 SNPs/indels (76%) were in introns, 35 (11%) in untranslated regions (UTRs), and 44 (13%) in coding sequence (CDS) regions (Fig. 6a). The subsequent analysis revealed that of the 44 alterations in CDS regions, 34 gave rise to non-synonymous substitutions within 33 genes, while ten led to synonymous substitutions in ten genes. CDS regions that resulted in non-synonymous substitutions were blasted with the annotated protein and homologous information of *Arabidopsis thaliana* using the TAIR10 database (the Arabidopsis Information Resource, https://www.arabidopsis.org/). Nineteen annotated genes that were found to have substitutions in the CDS regions are listed in Table 3. For sensitive analysis, the GSEA (Gene Set Enrichment Analysis) method was used to interpret the distinct phenotype differences [38]. Each gene was categorized by GO terms (Gene Ontology, https://www.geneontology.org/) at depth of three, which are shown in Fig. 6b. Based on the results of the GSEA analysis, nine annotated genes were categorized into two domains (molecular function and biological process) and were subdivided into seven GO terms (Fig. 6b). Most of the GO terms were found to be involved with molecular function and were specialized for purine nucleoside/nucleotide binding. In the biological process domain, Cre10g.446650 and Cre06.g297082 were identified as genes related with cell division and cell cycle regulation. It is speculated that continuous illumination over extended periods of time was the possible cause behind the mutations in those genes within CC-124H. These mutations could have caused alterations within the cell cycle of CC-124H, which could explain the faster growth under normal conditions and higher lipid accumulation under nutrient stress.

**Table 3:** CC-124H specific genomic DNA information of single polymorphisms (SNPs) and insertion/deletion (indels)

chr^a^	Position	Type	Genic	Feature	TAIR10.gene_ID^b^	TAIR10.des^c^
1	7296452	SNP	Cre01.g052650	CDS	AT3G01310.2	Phosphoglycerate mutase-like family protein
3	8957195	Indel	Cre03.g208833	CDS	AT1G35530.2	DEAD/DEAH box RNA helicase family protein
3	8972552	Indel	Cre03.g209281	CDS	AT1G08190.1	Vacuolar protein sorting 41
3	3163671	SNP	Cre03.g165215	CDS	AT5G45900.1	ThiF family protein
3	4595120	SNP	Cre03.g177007	CDS	AT5G63080.1	2-Oxoglutarate (2OG) and Fe(II)-dependent oxygenase superfamily protein
6	82060	Indel	Cre06.g249500	CDS	AT4G14140.1	DNA methyltransferase 2
6	262541	Indel	Cre06.g250850	CDS	AT3G24320.1	MUTL protein homolog 1
6	408975	Indel	Cre06.g252100	CDS	AT5G58410.1	HEAT/U-box domain-containing protein
6	1502181	Indel	Cre06.g259700	CDS	AT3G52570.1	Alpha/beta-Hydrolases superfamily protein
6	1804583	Indel	Cre06.g262500	CDS	AT4G24560.1	Ubiquitin-specific protease 16
6	2321106	SNP	Cre06.g267250	CDS	AT3G23610.3	Dual specificity protein phosphatase 1
6	5727069	SNP	Cre06.g286950	CDS	AT3G04710.1	Ankyrin repeat family protein
6	7089219	SNP	Cre06.g297082	CDS	AT5G07280.1	Leucine-rich repeat transmembrane protein kinase
9	6439357	Indel	Cre09.g407110	CDS	AT5G19840.2	2-oxoglutarate (2OG) and Fe(II)-dependent oxygenase superfamily protein
9	5152546	SNP	Cre09.g399030	CDS	AT1G06570.1	phytoene desaturation 1
9	5224176	SNP	Cre09.g399439	CDS	AT5G19080.1	RING/U-box superfamily protein
9	7001422	SNP	Cre09.g410950	CDS	AT1G37130.1	Nitrate reductase 2
9	7399225	SNP	Creg09.g413200	CDS	AT1G03740.1	Protein kinase superfamily protein
10	3763671	SNP	Cre10.g446650	CDS	AT3G27730.1	ATP binding; ATP-dependent helicases; DNA helicases
11	222386	Indel	Cre11.g467552	CDS	AT2G22480.1	Phosphofructokinase 5

^a^Chromosome number in *Chlamydomonas reinhardtii*

^b^Gene ID in *Arabidopsis thaliana* homologous with genes in *C. reinhardtii*

^c^Description of TAIR10.gene_ID

## Discussion

There are growing interests in microalgal strain development through genetic manipulation for enhancing their biomass and lipid productivity [39]. Moreover, the systems and methods used for large-scale outdoor cultivation of microalgae have been developed and tested with various microalgal species, and these efforts have elucidated the potential for economically feasible biofuel production from microalgae [40, 41]. Therefore, outdoor mass cultivation techniques coupled with strain development through genetic manipulation of metabolic networks may eventually be needed to realize a successful commercialization of microalgae-based biofuel production. Unfortunately, however, there are still constraints in the use of genetically modified microalgae in an outdoor cultivation system due to safety concerns [31, 42]. Some researchers have pointed out that the biosafety issue of GMOs should be addressed before they are introduced into the environment [42–44]. In this context, forward genetics approaches have been applied in the microalgae field to generate non-GMOs that show enhanced traits in terms of biomass and lipid production as well as stress tolerance [11, 28, 31, 45]. Moreover, it has been proposed that experimental evolution can be an alternative strategy to circumvent public misgivings about the cultivation of genetically modified microalgae in an outdoor system [42]. It is known that adaptive evolution caused by an accumulation of mutations conferring selective advantages against particular environment conditions can lead a new generation which possess novel traits [46–48].

In the present study, we isolated CC-124H which likely adapted to the laboratory conditions. Although this strain was not intentionally obtained through experimental evolution, the growth trait of the CC-124H strain based on the DCW is visibly distinct from its WT strain CC-124 compared to 5 years ago (Fig. 1), and the high biomass productivity is linked with the cell population of CC-124H containing relatively larger cells compared to the CC-124L (Additional file 2: Figure S1). Although CC-124H exhibited superior growth rate in terms of optical density and DCW compared to CC-124L, (Fig. 2) the difference in the growth profile between CC-124H and CC-124L is not as great in comparison to the difference between CC-124H and the ancestral CC-124 strain from 5 years ago. It should be noted that CC-124L is the CC-124 strain that has been kept for 5 years in slant culture tube under low light condition. Since CC-124L was under long-term storage in the slant and was rarely re-transferred into a new agar slant, it obviously underwent a far smaller number of division cycles in comparison to CC-124H, and it is thought to be genetically close to the ancestral CC-124. However, it is unlikely that CC-124L is genetically identical to its CC-124 ancestor, and this may explain the slightly enhanced performance of CC-124L compared to its ancestor.

Traditionally, *C. reinhardtii* has not been known as an oleaginous species [35]. On the other hand, it has also been reported that the average lipid content of *C. reinhardtii* is comparable to that of other microalgae species, and the natural level of lipid contents can widely vary even among *C. reinhardtii* strains [9, 41]. More recently, Nakanishi et al. suggested that *C. reinhardtii* JSC4 isolated from the ocean accumulates a significant amount of lipids indicating that it also has potential to be used as industrial strain for biofuel production [49]. Current studies on microalgal strain development have mainly focused on both biomass and lipid production, as the overall lipid productivity is based on these two factors. In general, the lipid content increases under nutrient stress conditions such as N or S starvation, while the growth rate declines in *C. reinhardtii* [12–14]. In fact, several studies have provided clues to elucidate the mechanism behind the lipid accumulation in response to N deprivation. N deprivation led to the downregulation of genes for components of photosynthesis and the upregulation of genes for fatty acid biosynthesis [50, 51]. In addition, a time course transcriptome analysis of *C. reinhardtii* revealed that the gene cluster involved in lipid metabolism was highly expressed during the lipid accumulation phase, whereas photosynthesis related genes which play a crucial role in cell proliferation were downregulated during the stationary phase [52]. In *Nannochloropsis* sp., which is currently considered as a model microalga for biofuel production, genes associated with energy metabolism were downregulated in response to N starvation [53, 54]. These imply that there is an inverse relationship between the expression patterns of genes responsible for lipid metabolism and cell proliferation, and thus growth and lipid accumulation events are rarely synchronized during microalgae cultivation [55–57].

In agreement with the previous studies, we observed that the FAME content of the CC-124L gradually increased in response to N or S deprivation, while the amount of biomass was reduced during the cultivation (Fig. 3a–c). Interestingly, however, CC-124H had a significantly enhanced FAME yield that was approximately 2.5-fold greater than that of CC-124L under N depleted conditions, and this was further verified through the lipid body staining assay and electron microscopy (Figs. 3b, 4). This amount of increase in FAME productivity is due to the improved growth trait as well as lipid accumulation even under N starvation conditions.

The analysis of the carbohydrate, protein, and chlorophyll contents likewise showed differences between the CC-124L and CC-124H. Under normal media, CC-124H had a nearly two fold higher carbohydrate content compared to the CC-124L, while the overall lipid contents were similar (Figs. 3a, 5a). This indicates that CC-124H assimilates carbon into carbohydrates more aggressively than that of the CC-124L, either through photosynthesis or through greater assimilation of acetate. Under S starvation conditions, mutant cells also showed higher levels of carbohydrate accumulation than that of the CC-124L. Accumulation of higher levels of starch granules have been reported in *C. reinhardtii* subjected to S depleted growth conditions [13, 14]. However, under N starvation conditions, CC-124H was shown to accumulate lipids to a much greater extent than that of the CC-124L, while its overall carbohydrate contents decreased. One possible explanation is that under stress conditions, carbohydrate synthesis is downregulated in favor of lipid synthesis in mutants [58, 59]. Another possibility is that the conversion and repartitioning of existing intracellular carbohydrates into fatty acid storage form may have a substantial contribution towards the observed increase in lipid contents [59, 60]. In addition, lower levels of protein content were found in CC-124H compared to the CC-124L under S depleted conditions. The lower percentage of proteins in the mutant cells can be directly attributed to the altered carbon partitioning and increased percentage of carbohydrates and lipids in the cells due to the fact that CC-124H has an elevated level of carbon fixation. Moreover, as mentioned previously, depletion of N and S in growth media has a profound effect on the metabolic pathways of microalgae. As expected, depletion of N in the media resulted in a substantial decrease in the overall protein content in both CC-124L and CC-124H. Reduction in the protein content is expected under N starvation due to the requirement of nitrogen in the synthesis of most amino acids, and as such cells typically enter cell cycle arrest while expressing higher levels of stress marker metabolites such as carbohydrates and lipids [13, 51].

Based upon all these observations, we assumed that CC-124H was modified through adaptive evolution during the maintenance of CC-124 under laboratory conditions. To further understand the enhanced traits of CC-124H in biomass and lipid production, we next performed SNPs/indels analysis which can identify non-synonymous substitutions at a molecular level. Our results revealed that many of the non-synonymous substitutions caused by SNPs/indels were found in genes required for cell cycle, division or proliferation. These included the ThiF family protein (Cre03.g165215), U-box and RING/U-box proteins (Cre06.g252100 and Cre09.g399439), ubiquitin-specific protease 16 (UBP16, Cre06.g262500), leucine-rich repeat receptor protein kinase (LRR-RPK, Cre06.g297082), DEAD/DEAH box RNA helicase family protein (Cre03.g208833), dual specificity protein phosphatase (Creg06.g267250) and ankyrin repeat family protein (Cre06.g286950) (Table 3).

Thiamin, known as vitamin B1, is an essential cofactor that is synthesized in microalgae as well as plants and bacteria. ThiF motifs located in ubiquitin-activating enzyme (E1) form a thiol-ester bond with ubiquitin to facilitate ubiquitination, which signals for proteasome-mediated degradation of a target protein [61]. Among the components of the ubiquitination systems, ubiquitin-ligase (E3) interacts with specific proteins. Higher plants are known to primarily possess three types of E3 ligase, which are RING, HECT, and U-box. Recent studies on ubiquitin systems in *C. reinhardtii* have involved various ubiquitin-mediated processes such as control of the circadian clock, disassembly of cilia and flagella, and lipid modulation [62, 63]. In this study, SNPs and indels were detected in the/U-box and RING/U-box proteins (Cre06.g252100 and Cre09.g399439), and it is predicted that mutations at these sites may be the cause behind the immobility and unique cell division characteristics of CC-124H. Moreover, ubiquitin-specific protease 16 (UBP16, Cre06.g262500), annotated as AT4G24560 in *A. thaliana*, was found in this study. UBP16 exhibits partial redundancy and is stabilized under salt stress conditions, and it is also known to be involved in cell proliferation [64, 65].

In addition, SNPs were detected at the multi-functional gene, leucine-rich repeat receptor protein kinase (LRR-RPK, Cre06.g297082). Leucine-rich repeat (LRR) motifs are conserved across many organisms and are known to interact with various proteins. The mutations in the LRR motifs may cause some phenotypic changes, especially in the reproductive cells [66]. In *A. thaliana*, a male EMS1 mutant encoding LRR-RPK had no cytokinesis of microspores [67].

The DEAD/DEAH box RNA helicase family protein (Cre03.g208833) is also proposed to be involved in cellular growth and division and has an important role in unwinding nucleic acids. In the case of *Prorocentrum donghaiense*, proteomics analysis shows that the DEAD/DEAH box protein was upregulated during proliferation [68]. Dual specificity protein phosphatases (DUSP) are responsible for the dephosphorylation of serine, threonine and tyrosine and are also involved in MAP kinase signaling cascade. They can also be involved in the cell cycle, as downregulation of DUSP are linked to tumor proliferation in mammals [69, 70]. Lastly, the ankyrin repeat family protein (Cre06.g286950) regulates many intracellular processes such as signal transduction and cell cycles [71]. The SNPs in the cell cycle related genes are thought to have resulted in substantial morphological changes in CC-124H. Indeed, CC-124H showed increased proportion of the cells in the palmella stage of development, and lacked flagella which resulted in general loss of mobility compared to the wild-type cells.

While CC-124H accumulated much higher lipid contents than CC-124L, surprisingly, there were no SNPs in the coding region of the genes directly involved in the lipid synthesis. One possibility is that the mutations in the cell cycle machinery of the organism have indirectly led to the changes in the lipid accumulation. The above mentioned palmelloid phenotype in CC-124H is thought to have originated due to the irregular cell cycle. A previous study has found that cells with unregulated cell division such as tumor cells often show high lipid contents [72–74]. In addition, it should be noted that there were hundreds of SNPs that occurred in the non-coding regions of the genome such as promoter, terminator, and introns. Most of these could not be identified and were not included in the results of this study. While the SNPs in the coding region often results in loss of function mutations which is deleterious to the survival of the organism, SNPs in the non-coding regions do not cause such loss of function mutations, which allows the organism to retain and accumulate these in far greater numbers. However, the SNPs in the non-coding regions can result in substantial changes in the expression level of the individual genes, and multiple changes such as these can have global effects on the overall systems biology of the organism. In addition, it is quite probable that there were SNP’s in the UTR of lipid synthesis related genes such as diacylglycerol acyltransferase, plastid-lipid associated protein PAP, and pyruvate dehydrogenase kinase which directly resulted in the expression level of these genes. It is thought that all of these changes could have resulted in substantial alterations in the overall lipid synthesis in the organism.

Since the initial study by Goto et al., many studies have revealed that photoperiodic cell division is mediated by the circadian clock, and mRNA abundances are oscillated during the circadian cycle in the unicellular photosynthetic algae, *Chlamydomonas* and *Ostreococcus* [75–78]. More recently, additional research has reported that the *C. reinhardtii* strain CC-503 evolved for 1880 generations under a continuous light condition had 149 SNPs resulting in non-synonymous amino acid substitutions with a 35% greater growth rate compared to its progenitor population [26]. These results indicate that several physiological events in CC-124H including cell proliferation timing and frequency are possibly adapted to gain selective advantages under a continuous illumination condition, although the underlying mechanism remains undiscovered in this study.

## Conclusion

In the present study, we isolated the *C. reinhardtii* CC-124H strain whose growth trait was distinct from its ancestral WT, and further demonstrated that CC-124H exhibited significantly increased amount of biomass and lipid production compared to the CC-124L. Moreover, an integrated genome wide analysis allowed us to identify sequence variations within individual genomes which may result in the phenotypic changes of CC-124H. These findings suggest that the concept of adaptive evolution combined with genome wide analysis can be used as an alternative strategy for the development of microalgal strains that meet academic and industrial standards for biofuel production.

## Methods

### Microalgal strain, culture conditions and isolation of CC-124H

*Chlamydomonas reinhardtii* CC-124(−) strain was purchased from the Chlamydomonas Resource Center at the University of Minnesota (St. Paul, MN, USA; http://chlamycollection.org/). Cells were maintained on Tris-acetate-phosphate (TAP) agar medium [79] consisting of 2.42 g/L Tris, 0.375 g/L NH_4_Cl, 0.1 g/L MgSO_4_·7H_2_O, 0.05 g/L CaCl_2_·2H_2_O, 0.0108 g/L K_2_HPO_4_, 0.0054 g/L KH_2_PO_4_, 1 mL/L glacial acetic acid and 1 mL/L Hutner’s trace elements (50 g/L Na_2_EDTA·2H_2_O, 22 g/L ZnSO_4_·7H_2_O, 11.4 g/L H_3_BO_3_, 5.06 g/L MnCl_2_·4H_2_O, 1.61 g/L CoCl_2_·6H_2_O, 1.57 g/L CuSO4·5H_2_O, 1.10 g/L (NH_4_)_6_Mo_7_O_24_·7H_2_O, and 4.99 g/L FeSO_4_·7H_2_O at 25 °C under continuous illumination of 50 μmol/m^2^/s. Cells were transferred onto fresh TAP agar plate once every month for 5 years. During the maintenance of *C. reinhardtii* CC-124(−), a cell representing rapid growth, CC-124H, was isolated. Both the microalgal strains, *C. reinhardtii* CC-124(−) and CC-124H were cultivated in 500 mL volumetric flasks with 200 mL working volumes at 25 °C with shaking (120 rpm) under fluorescent light (120 µmol/μmol/m^2^/s).

### Growth and nutrient analysis

CC-124L and CC-124H strains were cultivated under normal conditions (TAP medium) as described above: 25 °C, 120 rpm and 120 μmol/m^2^/s fluorescent light. For the N and S starvation, stationary phase cells were harvested and washed twice with TAP medium, and then, the cells (OD_750_: ~ 0.7) were inoculated into TAP medium without N and S, respectively. Cell growth was determined by measuring the cell density (in cells/mL), optical density (OD) and dry cell weight (DCW). Cell density was counted with a cell counter (Cellometer AutoT4™, Nexcelom Bioscience, USA), and the OD_750_ was measured by a UV/Vis spectrometer (Shimadzu Co., Japan) at the indicated time points, and the DCW was estimated by filtering the cells with GF/C filter paper (Whatman, USA), drying at 120 °C overnight, and weighing on a microbalance (CP224S, Sartorious, Germany). The specific growth rate of the microalgae based on the DCW was calculated with the following equation:

$${\text{Specific growth rate }}\left( {\mu /{\text{h}}} \right) = { \ln }{{\left( {{{X_{ 2} } \mathord{\left/ {\vphantom {{X_{ 2} } {X_{ 1} }}} \right. \kern-0pt} {X_{ 1} }}} \right)} \mathord{\left/ {\vphantom {{\left( {{{X_{ 2} } \mathord{\left/ {\vphantom {{X_{ 2} } {X_{ 1} }}} \right. \kern-0pt} {X_{ 1} }}} \right)} {\left( {t_{ 2} - t_{ 1} } \right)}}} \right. \kern-0pt} {\left( {t_{ 2} - t_{ 1} } \right)}},$$

where *X*_1_ and *X*_2_ are the initial and final DCW, and *T*_1_ and *T*_2_ are the initial and final times.

The concentrations of ammonium (NH_4_^+^), (phosphate (PO_4_^3−^), sulfate (SO_4_^2−^) and acetate (CH_3_CO_2_^−^) in the broth were determined by ion chromatography (881 compact IC pro, Metrohm, Swiss) with a Metrosep C4 150 column, Metrosep A Supp5 150 column and Metrosep Organic Acids 250 column for cations, anions and organic acids, respectively.

### Fatty acid methyl esters (FAMEs) analysis by gas chromatography (GC)

Microalgal cells were harvested by centrifugation at 5035×*g* for 10 min. (Supra-22K, Hanil Science Industrial, Republic of Korea). The pellets were washed twice with deionized water, lyophilized at − 80 °C for 3 days and then used for lipid extraction. For the lipid extraction, a chloroform–methanol mixture (2:1, v/v) was added to 10 mg of lyophilized cells and then vigorously mixed in a Teflon-sealed screw-capped Pyrex tube (Pyrex, USA) for 10 min. 0.5 mg of heptadecanoic acid (C17:0) were added as an internal standard for gas chromatography (GC). For transesterification which converts extracted lipid into fatty acid methyl esters (FAMEs), 1 mL of methanol and 300 μL of sulfuric acid were added and then incubated at 100 °C for 20 min. After cooling down, 0.3 N concentration of sodium hydroxide was added to rinse the remaining methanol and sulfuric acid. After centrifugation at 4000 rpm for 10 min at room temperature, the organic phase (lower layer) was filtered using a 20 μm RC-membrane syringe filter (Sartorius Stedim Biotech, Germany). FAMEs were analyzed by GC (HP5890, Agilent, USA) equipped with a flame ionized detector (FID) and an HP-INNOWAX polyethylene glycol column (30 m × 0.32 mm × 0.5 μm; H19091 N-213, Agilent, USA). The FAME composition and content were determined with a 37-component mix of FAME standards (F.A.M.E. Mix C8–C24, Supelco, USA).

### Biochemical component analysis: total carbohydrates and proteins

To determine the biochemical components, 5 mg of a lyophilized biomass were required. Samples were reacted with anthrone reagents in a sulfuric acid/deionized water mixture (3:1, v/v) at 100 °C for 15 min. After cooling on ice, the absorbance was measured at 620 nm and calibrated with glucose at concentration of 0–240 mg/L as a standard [80]. To measure the protein content, the Quick Start™ Bradford Protein Assay (Biorad, USA) was used. The biomass was reacted with 1 N sodium hydroxide solution at 100 °C for 10 min. Then, the disrupted cells were reacted with 1× dye reagent for 5 min., and then, the absorbance was measured at 595 nm and calibrated with BSA standards.

### Chlorophyll fluorescence

Chlorophyll contents of *C. reinhardtii* were measured with methanol extraction using the following protocol [81]. 5 mL cultures of cells were harvested at 5035×*g* for 10 min and washed twice with deionized water. The pellets were resuspended in a same volume of methanol and incubated in a dark condition at 4 °C for 1 h. After centrifugation at 5035×*g* for 10 min, the absorbance of the supernatant was measured at 652 and 665 nm. The concentrations of chlorophyll a and b were calculated using the following equations.

$${\text{Chl a }}\left( {\upmu{\text{g}}/{\text{mL}}} \right) = - \; 8.0 9 6 2 \times {\text{OD}}_{{ 6 5 2\;{\text{nm}}}} + 1 6. 5 1 6 9 \times {\text{OD}}_{{ 6 6 5\;{\text{nm}}}}$$

$${\text{Chl b }}\left( {\upmu{\text{g}}/{\text{mL}}} \right) = 2 7. 4 40 5 \times {\text{OD}}_{{ 6 5 2\;{\text{nm}}}} - 1 2. 1 6 8 8 \times {\text{OD}}_{{ 6 6 5\;{\text{nm}}}}$$

Lastly, the chlorophyll concentration was normalized with the cell density using Cellometer AutoT4 (Nexcelom Bioscience, USA).

### Transmission electron microscopy (TEM)

Cells were centrifuged at 1644×*g* for 5 min and washed twice with 0.1 M phosphate buffer. Then, 0.1 M phosphate buffer containing 2.5% glutaraldehyde was used for fixation of each strain. After treatment with the Epon 812 embedding medium, specimens were observed under a bio-Transmission electron microscopy (Tecnai G2 Spirit, FEI Co., Netherlands, Installed at Korea Basic Science Institute).

### BODIPY staining and confocal microscopy

For detection of lipid bodies, cells were stained by BODIPY^®^ 505/515 (1 μM, 4,4-difluoro-1,3,5,7-tetramethyl-4-bora-3a,4a-diaza-*s*-indacene, Invitrogen, USA) solubilized in 0.2% dimethyl sulfoxide (DMSO) and incubated under a dark condition for 10 min. BODIPY stained cells were observed using a laser scanning confocal microscope (LSM 510 META NLO, Carl Zeiss, Germany) with the appropriate excitation and emission laser units (Argon 488 nm; 30 mW for excitation and HeNe 543 nm; 1 mW for emission). Images were taken with a 50× magnification, and image processing was done with the LSM Image Browser (Carl Zeiss, Germany).

### Genomic DNA preparation for DNA sequencing

Genomic DNA was extracted by a modified version of the standard phenol/chloroform extraction method [82]. Briefly, 5 mL of exponentially growing *C. reinhardtii* cells in TAP medium were harvested for genomic DNA extraction. Pellets were washed with 50 mM EDTA until no clumps were visible. After centrifugation at 7000 rpm for 10 min, 150 μL of distilled water and 300 μL of SDS-EB were added to the pellets. Subsequently, both 250 μL of phenol and chloroform were added and vortexed for 5 min. After centrifugation, the supernatants were separated, and the previous steps were repeated with the treatment of phenol and chloroform. For DNA precipitation, 2 volumes of pure ethanol were added and then kept at − 20 °C for 20 min and then centrifuged. Finally, DNA pellets were solubilized with an appropriate volume of TE buffer (1 mM Tris–HCl and 0.2 mM EDTA, both of pH 8).

### Whole genome sequencing and SNPs/indels analysis

A schematic workflow for the genome sequencing and SNPs/indels analysis was presented in Additional file 3: Figure S2. Briefly, DNA libraries and cluster construction were performed using TruSeq DNA PCR free sample Preparation Kit (Illumina, USA), TruSeq Rapid SBS kit (Illumina, USA) and TruSeq Rapid PE Cluster kit (Illumina, USA). The *C. reinhardtii* DNA libraries were sequenced using Illumina HiSeq 2500 (Illumina, USA). The sequencing data were submitted to the NCBI Sequence Read Archive (SRA) under accession code SRP113748. A paired-end sequencing platform was used to analyze the SNPs/indels between CC-124H and CC-124L by Illumina HiSeq 2500. Short read sequences from CC-124 and CC-124H were trimmed by the SolexaQA package with the sequence quality and length trimming standard (Probability value: 0.05, phred quality score: 20, and minimum read length: 25 bp). Then, each sequencing dataset was mapped to the *C. reinhardtii* reference genome v5.5 (Phytozome ver 10.1; http://www.phytozome.net/) using the Burrows-Wheeler Aligner (BWA). The raw SNPs/indels detection and extraction of consensus were done with SAMtools and In-house script. The detected SNPs/indels were filtrated with the following criteria: read depth: ≥ 3; mapping quality: ≥ 30 and biallelic SNPs/indels. Unmapping regions were eliminated in the case of unknown nucleotides. Finally, the SNPs/indels were classified and annotated by Pfam (http://pfam.sanger.ac.uk/), Panther (http://www.pantherdb.org), KOG (http://www.ncbi.nlm.nih.gov/COG/), KEEG (http://www.genome.jp/kegg/), and GO (http://www.geneontology.org/) analysis.

## Additional files

**Additional file 1: Table S1.** Specific growth rate of CC-124 in year 2010, 2013, and 2015.

**Additional file 2: Figure S1.** Cell size distribution of CC-124L and CC-124H in liquid TAP medium.

**Additional file 3: Figure S2.** Flow chart of the genome wide SNPs/indels analysis method.

**Additional file 4.** List of genome data of single nucleotide polymorphisms (SNPs) and insertion-deletions (indels) comparison of CC-124L and CC-124H.

## Abbreviations

WT: wild-type
N: nitrogen
S: sulfur
TAG: triacylglycerol
OD: optical density
FAME: fatty acid methyl ester
SNPs: single nucleotide polymorphisms
indels: insertion/deletions
GMO: genetically modified organism
NGS: next-generation sequencing
TEM: transmission electron microscope
DCW: dry cell weight
TAP: Tris-acetate-phosphate
