# Supplementary material for: Isolation, phenotypic characterization and genome wide analysis of a Chlamydomonas reinhardtii strain naturally modified under laboratory conditions: towards enhanced microalgal biomass and lipid production for biofuels
Source: Biotechnol Biofuels. 2017 Dec 22;10:308. doi: 10.1186/s13068-017-1000-0 (PMC5740574; doi:10.1186/s13068-017-1000-0)
Supplement: Supplementary file 1 — Additional file 1: Table S1. Specific growth rate of CC-124 in year 2010, 2013, and 2015. [file 13068_2017_1000_MOESM1_ESM.docx]

**Table S1. Specific growth rate of *C. reinhardtii* in year 2010, 2013, and 2015.**

| **Year** | **Specific growth rate (mg·mL^-1^·h^-1^)^a^** | |
| --- | --- | --- |
|  | 0 – 24 h | 24 – 72 h |
| 2010 | 0.023 ± 0.008 | 0.031 ± 0.004 |
| 2013 | 0.046 ± 0.004 | 0.029 ± 0.000 |
| 2015 | 0.085 ± 0.007*** | 0.010 ± 0.000 |

Data are expressed as ± SD (n = 3). Significant differences, as determined by Student’s *t* test, are indicated by asterisk (**P* < 0.05, ***P* < 0.01, ****P* < 0.001).

^a^ Specific growth rate based on dry cell weight was calculated as following equations:

Specific growth rate (μ/day) = ln(X_2_/X_1_)/(T_2_ - T_1_) where X_1_ and X_2_ are the initial and final dry cell weight, and T_1_ and T_2_ are the initial and final times.
