# Supplementary figures and images for: Isolation, phenotypic characterization and genome wide analysis of a Chlamydomonas reinhardtii strain naturally modified under laboratory conditions: towards enhanced microalgal biomass and lipid production for biofuels
Source: Biotechnol Biofuels. 2017 Dec 22;10:308. doi: 10.1186/s13068-017-1000-0 (PMC5740574; doi:10.1186/s13068-017-1000-0)

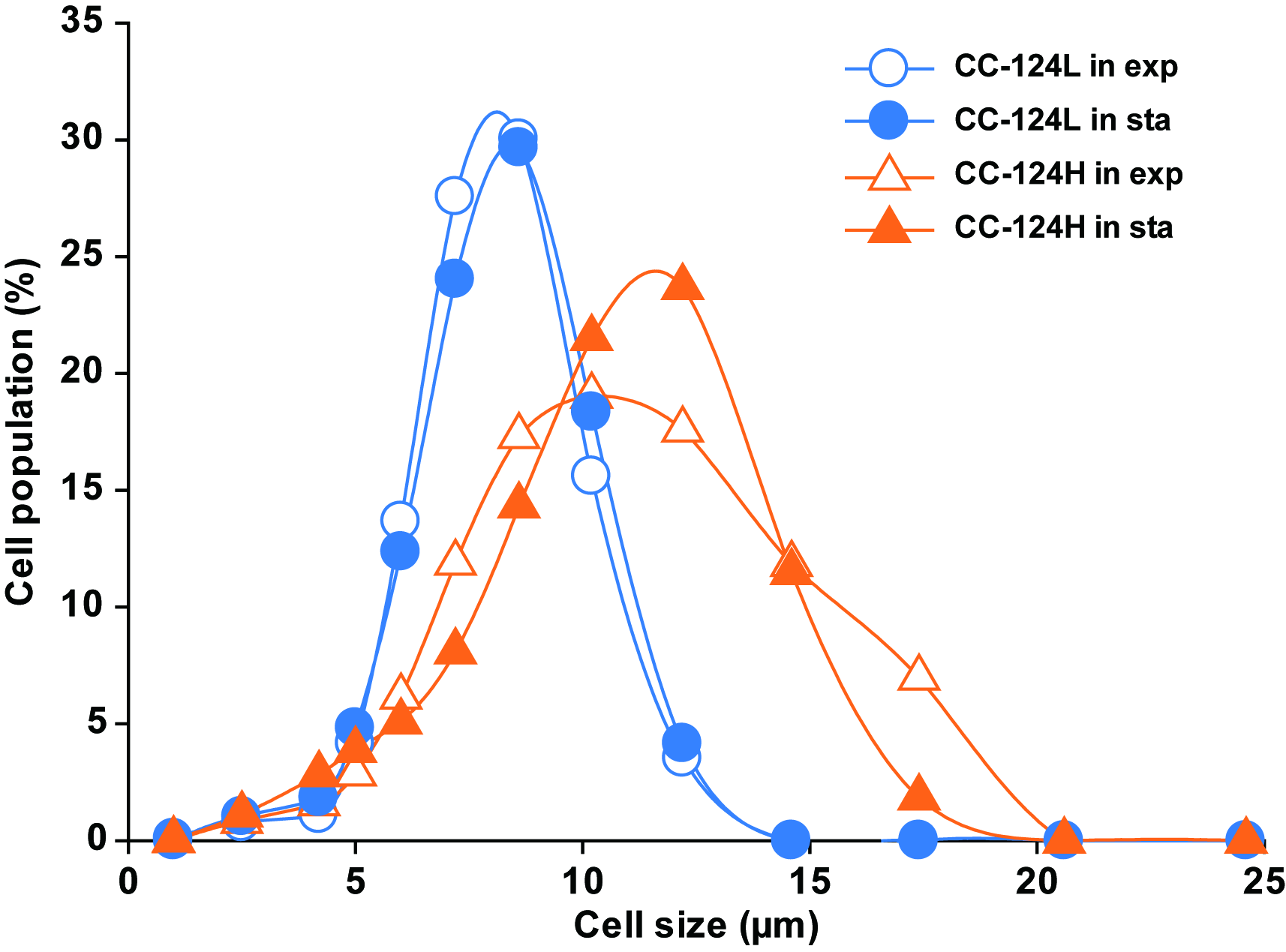

Supplement: Supplementary file 2 — Additional file 2: Figure S1. Cell size distribution of CC-124L and CC-124H in liquid TAP medium. [file 13068_2017_1000_MOESM2_ESM.tif]

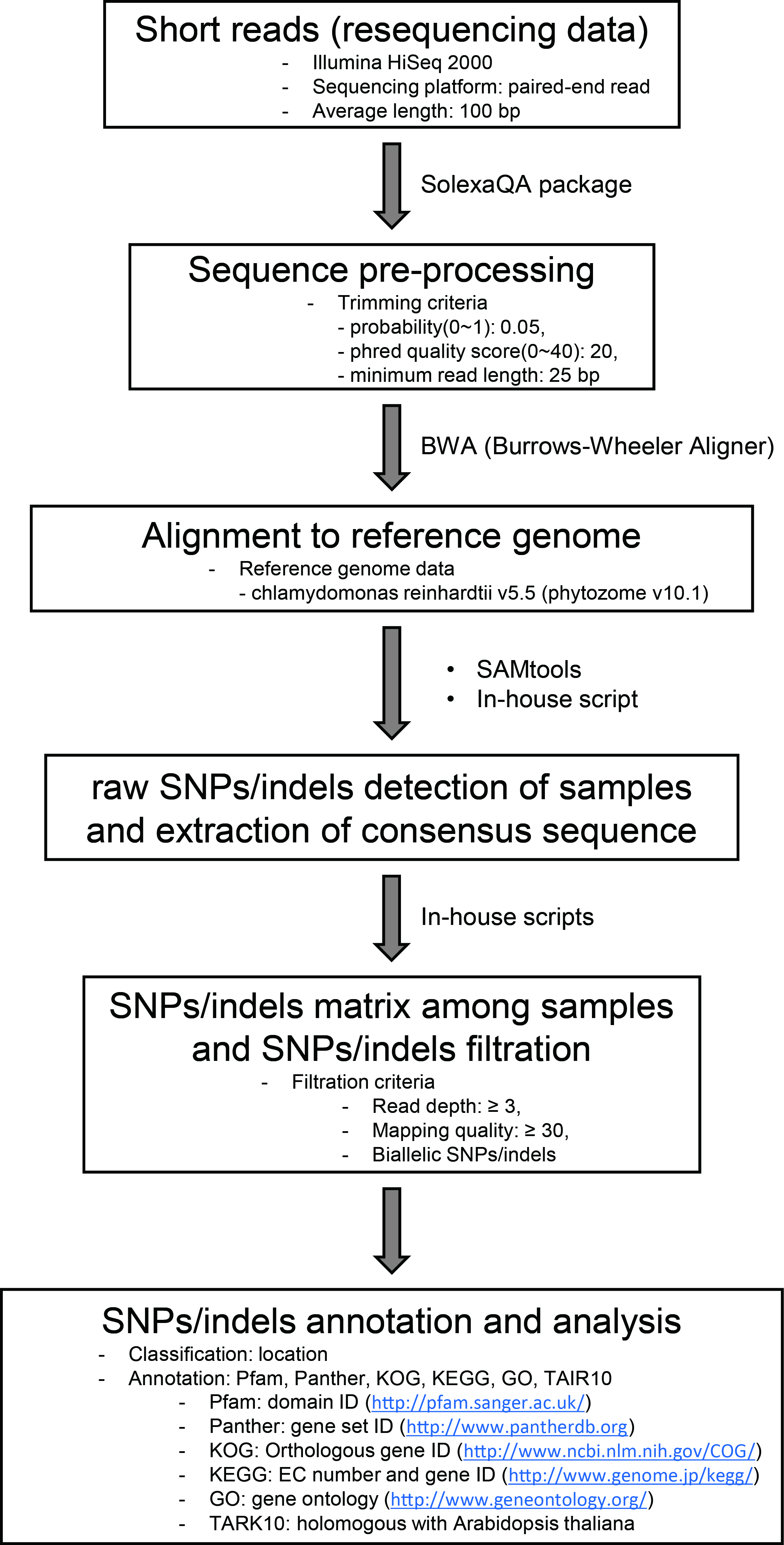

Supplement: Supplementary file 3 — Additional file 3: Figure S2. Flow chart of the genome wide SNPs/indels analysis method. [file 13068_2017_1000_MOESM3_ESM.tif]
